# Evaluation of Relapse-Free Survival in T3N0 Colon Cancer: The Role of Chemotherapy, a Multicentric Retrospective Analysis

**DOI:** 10.1371/journal.pone.0080188

**Published:** 2013-12-05

**Authors:** Roberta Grande, Domenico Corsi, Raffaello Mancini, Donatello Gemma, Fabrizio Ciancola, Isabella Sperduti, Lorena Rossi, Agnese Fabbri, Maria G. Diodoro, Enzo Ruggeri, Germano Zampa, Sara Bianchetti, Teresa Gamucci

**Affiliations:** 1 Medical Oncology Unit–ASL Frosinone, Italy; 2 S. Giovanni Calibita Hospital–Fatebenefratelli Isola Tiberina, Rome, Italy; 3 Department of Surgery, Regina Elena Cancer Institute, Rome, Italy; 4 Bio-Statistics Unit, Regina Elena National Cancer Institute, Rome, Italy; 5 Medical Oncology Unit, Belcolle Hospital, Viterbo, Italy; 6 UOSA Oncologia ASL RM/A, Rome, Italy; 7 Medical Oncology Unit, Regina Apostolorum, Albano Laziale, Rome, Italy; 8 Department of Pathology, Regina Elena National Cancer Institute, Rome, Italy; Ospedale Pediatrico Bambino Gesù, Italy

## Abstract

**Background:**

Adjuvant chemotherapy (AC) in Stage II Colon Cancer (CC) is still under debate. Choice should be based on patients and disease characteristics. According to guidelines AC should be considered in high-risk T3N0 patients. No data are available for better option in low-risk patients. The aim of the study is to retrospectively evaluate relapse-free survival (RFS) and disease-free survival (DFS) according to treatment received in T3N0 CC.

**Methods:**

RFS and DFS are evaluated with Kaplan-Meier method. Multivariate Cox proportional hazard model was developed using stepwise regression, enter limit and remove limit were p = 0.10 and p = 0.15, respectively.

**Results:**

834 patients with T3N0 CC were recruited. Median age was 69 (29–93), M/F 463/371, 335 low-risk patients (40.2%), 387 high-risk (46.4%), 112 unknown (13.4%); 127 (15.2%) patients showed symptoms at diagnosis. Median sampled lymph nodes were 15 (1–76); 353 (42.3%) patients were treated with AC. Median follow up was 5 years (range 3–24). The 5-years RFS was 78.4% and the 5-years DFS was 76.7%. At multivariate analysis symptoms, lymph nodes, and adjuvant chemotherapy were prognostic factors for RFS. AC is prognostic factor for all endpoints.

In low-risk group 5-years RFS was 87.3% in treated patients and 74.7% in non-treated patients (p 0.03); in high-risk group was respectively 82.7% and 71.4% (p 0.005).

**Conclusions:**

Data confirmed the role of known prognostic factors and suggest the relevance of adjuvant chemotherapy also in low-risk stage II T3N0 CC patients. However, the highest risk in low-risk subgroup should be identified to be submitted to AC.

## Introduction

Prognosis of radically resected colon cancer is determined by the pathologic stage of the disease at the diagnosis and the most important determinant of survival is the presence of metastases to regional lymph nodes. In stage II disease there is no involvement of regional lymph nodes or distant sites and after surgical resection the overall survival is approximately 70–80% at 5 years [1]–[5].

The goal of the adjuvant chemotherapy after curative resection of early-stage colon cancer is to remove microscopic local or metastatic disease in order to reduce the risk of tumor recurrence and to improve survival rate.

The role of the adjuvant chemotherapy in stage II colon cancer is still under debate [6]–[15], [19]–[23]. According to the published guidelines adjuvant chemotherapy is recommended in high-risk stage II patients identified by the presence of poor prognostic features including T4 tumors, poor histologic grade (grade 3 or 4 lesions), vascular (venous/lymphatic) or perineural invasion, obstruction or bowel perforation at initial diagnosis and less than 12 analysed (retrieved) lymph nodes) [1]; [5]–[12], [24], [28]. However, these recommendations have never been validated in the setting of a prospective clinical trial.

There is not an unequivocally accepted option for patients with stage II without these poor prognostic factors; decision on adjuvant treatment must be based on thorough discussion with the patient on an individual basis taking into account cancer features and patient characteristics: a treatment with fluoropyrimidines or the only observation could be proposed and the enrollment in clinical trials should be considered.

It is therefore important to identify the patients with stage II disease without poor prognostic factors who can benefit from adjuvant chemotherapy.

In this multicenter study we retrospectively evaluated the relapse-free survival (RFS) and disease-free survival (DFS) related to treatment in patients with stage II T3N0 colon cancer.

## Patients and Methods

From February 1990 to August 2011, 834 patients with stage II T3N0 colon cancer treated with fluoropyrimidine based adjuvant chemotherapy or surgery alone with at least a 3-years follow up were recruited from six Italian centres. Patients did not receive preoperative radiotherapy or chemotherapy. The follow up examination period exceeded 5 years in all centres; patients were monitored every three months for the first 3 years, every six months for the next two years and then annually. Data concerning treatments, recurrence and prognosis were retrospectively collected. According to the presence of the aforementioned prognostic features patients were divided into two groups: high risk [poor histologic grade (grade 3 or 4 lesions), vascular (venous/lymphatic) or perineural invasion, bowel obstruction at initial diagnosis and less than 12 analysed (retrieved) lymph nodes] and low risk stage II colon cancer. We obtained approval from our ethics committee (Ethics Committee, ASL Frosinone) and received a formal written consent. Patients provided written informed consent.

### Statistical analysis

Descriptive statistics was used to describe the patient characteristics. The receiver operating characteristics (ROC) analysis and Maximally Selected Rank Statistics were performed in order to find possible optimal cut-offs of the lymph nodes capable of splitting patients into groups with different outcomes probabilities. The Hazard risk and the confidence limits were estimated for each variable using the Cox univariate model. Significance was defined at the p≤0.05 level. A multivariate Cox proportional hazard model was also developed using stepwise regression (forward selection) by selecting those predictive variables that were significant upon univariate analysis. Enter limit and remove limit were p = 0.10 and p = 0.15 respectively. Kaplan-Meier method was used to estimate survival curves and differences between subgroups them was assessed by the log-rank test. All significance was defined at the p<0.05 level. Significance was defined at the p≤0.05 level. SPSS software (version 18.0, SPSS Inc., Chicago, Illinois, USA), R-Software (version 2.6.1) and MedCalc® (10.0.1) were used for all statistical evaluations.

## Results

Patient characteristics are listed in Table 1. We identified 335 patients with low risk and 387 with high risk; for 112 patients it was not possible to define the group of risk because of lack of some histologic features.

**Table 1:** Patient Characteristics (N = 834).

Category	Subcategory	N° of patients (%)
Median age (range)		69 (29–93)
Age under 80		757 (90.8)
Median follow up (range)		5 years (3–20)
Sex	Men	463 (55.5)
	Women	371 (44.5)
Tumor location	Distal	432 (51.8)
	Proximal	402 (48.2)
Grading	1	44 (5.3)
	2	612 (73.4)
	3	144 (17.3)
	Unknown	34 (4.0)
Risk group	Low	335 (40.2)
	High	387 (46.4)
	Unknown	112 (13.4)
Symptoms at diagnosis	Absence	707 (84.7)
	Bowel obstruction	44 (5.3)
	Bleeding/Anaemia	63 (7.6)
	Abdominal pain	20 (2.4)
Median sampled lymph nodes (range)		15 (1–76)
Treatment	Surgery alone	481(57.7)
	Surgery+Adjuvant Chemotherapy	353 (42.3)

The median follow up was 5 years (range 3–20). Recurrence of disease was appreciated in 138 patients: 44 patients with low and 68 with high risk respectively; 21 (2.5%) patients developed a second primary cancer.

Survival analysis was performed only for patients younger than 80 and with at least a 3-years follow up from surgery (85%).

The 5-year relapse-free survival (RFS) was 78.4% and the 5-year disease-free survival (DFS) was 76.7%.

The 5-year RFS in the low risk group was 81.1% and 77.4% in the high risk group (p 0.06), respectively.

The analysis of relapse-free survival related to the selected prognostic factors evidenced that the 5-years RFS was 65.4% for patients that presented bowel obstruction at diagnosis and 79.2% for those without symptoms or with minor symptoms (p 0.002).

The relapse-free survival related to sampled lymph nodes was 84.8% for a number of nodes >16 and 74.7% for a number <16 (p 0.04).

**Figure 1:**
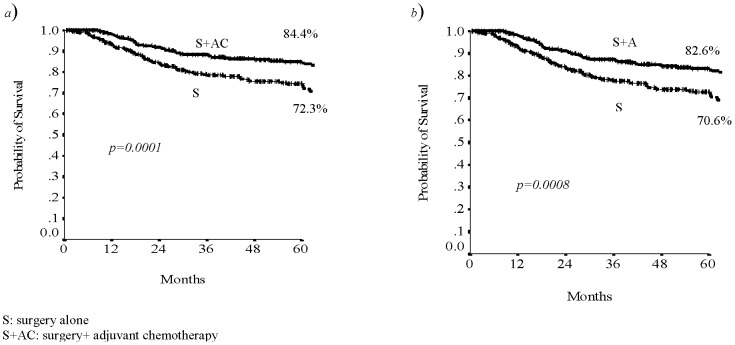
The 5-years RFS (a) and DFS (b) related to treatment.

Analysing the patient's data in relationship with the treatment received, we found that patients who underwent to surgery and adjuvant chemotherapy had a benefit in terms of RFS and DFS (p 0.0001 and p 0.0008, respectively; Fig. 1).

**Figure 2:**
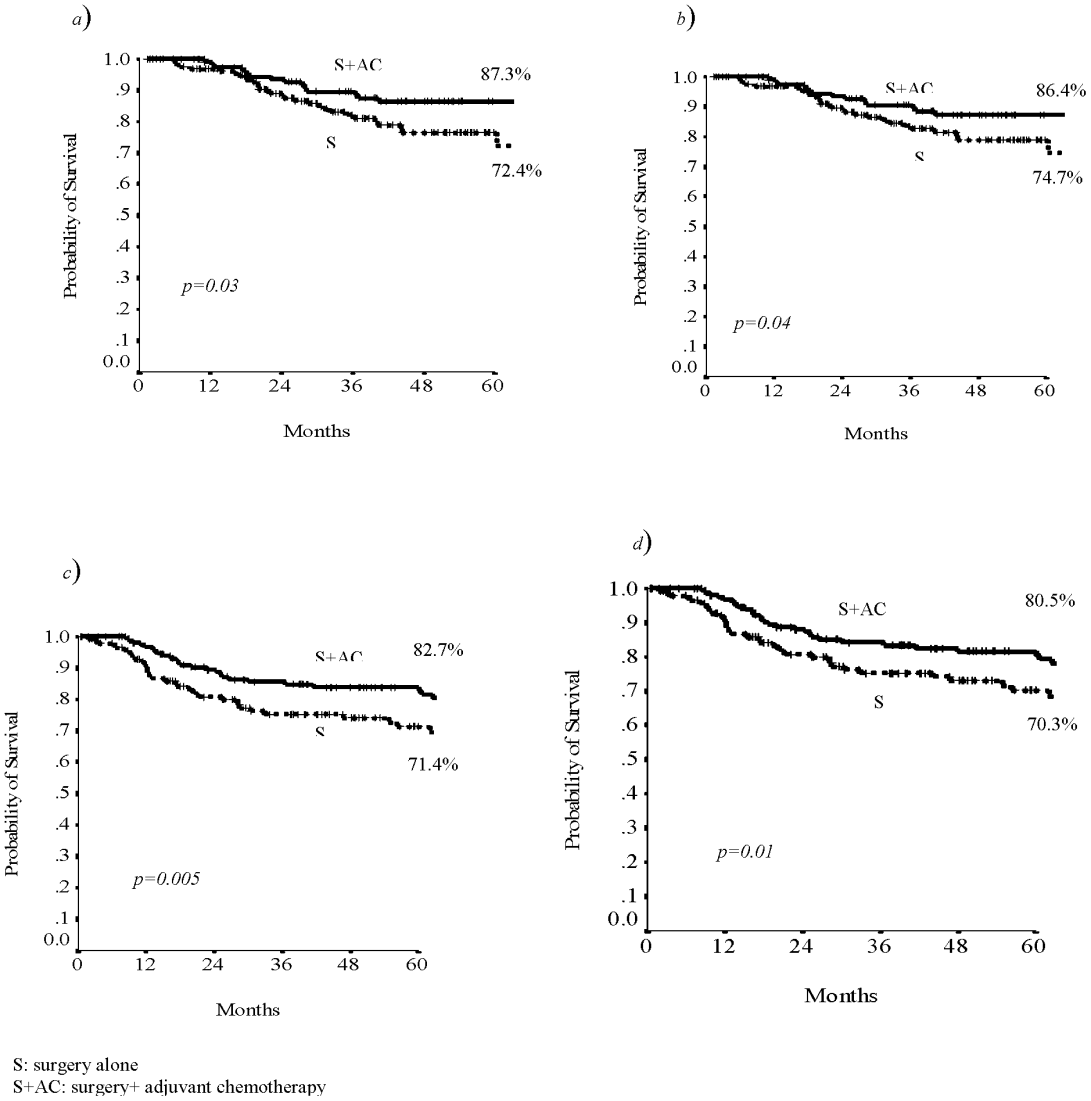
The 5-years RFS and DFS related to treatment in low risk group (a,b) and in high risk group (c,d).

The analysis for subgroup evidenced that in low risk group 5-years RFS was 87.3% in treated patients and 74.7% in non-treated patients (p 0.03; Fig. 2a) whereas in high risk group was respectively 82.7% and 71.4% (p 0.005; Fig. 2c).

In low risk group the disease-free survival was 86.4% in treated patients and 72.4% in patients who underwent to surgery alone (p 0.04; Fig. 2b). In high risk group the DFS was respectively 80.5% and 70.3% (p 0.01; Fig. 2d).

**Figure 3:**
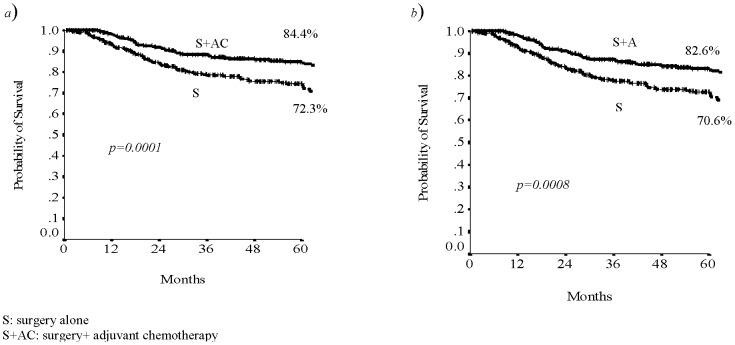
The 5-years overall survival curves related to treatment in low risk group.

The 5-years survival rate of all patients with T3N0 stage II colon cancer was 89.1%. In low risk group the overall survival rate was 98.7% for patients treated with adjuvant chemotherapy and 89.7% for patients treated with surgery alone (p = 0.003; Fig. 3). In high risk group the overall survival rate was 92.7% and 88.8% for patients treated with chemotherapy and surgery alone, respectively (p = 0.04).

At univariate analysis sex, age, site, symptoms, sampled lymph nodes and treatment were statistically significant (Table 2).

**Table 2:** Univariate analysis.

Variables	DFS		RFS		OS	
	HR (CI 95%)	*p*	HR (CI95%)	*p*	HR (CI95%)	*p*
BO vs no S	2.50 (1.47–4.26)	0.001	2.76 (1.62–4.72)	<0.0001	1.98 (0.79–4.95)	0.15
>68 yr vs <68 yr	1.50 (1.09–2.06)	0.01	1.46 (1.04–2.04)	0.03	2.02 (1.20–3.40)	0.008
M vs F	1.48 (1.07–2.04)	0.02	1.40 (1.0–1.96)	0.05	1.63 (0.95–2.78)	0.08
No AC vs AC	1.80 (1.31–2.49)	<0.0001	1.96 (1.39–2.76)	<0.0001	2.46 (1.39–4.33)	0.002
Distal vs Proximal	1.57 (1.11–2.21)	0.01	1.72 (1.20–2.47)	0.003	1.41(0.81–2.45)	0.22
LN <16 vs >16	1.47 (1.01–2.12)	0.04	1.58 (1.07–2.35)	0.02	1.57 (0.80–3.09)	0.19
Grading 3 vs 1–2	1.05 (0.68–1.60)	0.84	1.09 (0.70–1.69)	0.71	1.69 (0.72–3.94)	0.23

BO: bowel obstruction; S: Symptoms; M: male; F: female; AC: adjuvant chemotherapy; LN: lymph nodes; yr: year.

At multivariate analysis the only statistically significant variables are shown in Table 3.

**Table 3:** Multivariate analysis.

Variables	DFS		RFS		OS	
	HR (CI 95%)	*p*	HR (CI95%)	*p*	HR (CI95%)	*p*
BO vs no S	2.63 (1.46–4.74)	0.001	2.87 (1.58–5.20)	0.001	-	ns
>68 yr vs <68 yr	-	ns	-	ns	1.64 (0.94–2.88)	0.08
M vs F	1.52 (1.04–2.22)	00.03.00	1.51 (1.01–2.25)	0.04	16.7 (0.96–2.93)	0.07
No AC vs AC	2.06 (1.41–4.74)	<0.0001	2.30 (1.54–3.45)	<0.0001	2.15 (1.19–3.86)	0.01
Distal vs Proximal	1.52 (1.04–2.23)	0.03	1.72 (1.14–2.59)	0.01	-	ns

BO: bowel obstruction; S: Symptoms; M: male; F: female; AC: adjuvant chemotherapy, ns: not statistical significant;yr:year.

## Discussion

Even though adjuvant chemotherapy is the standard of care in stage III colon cancer, its routine use in patients with stage II colon cancer is still controversial [1]; [3]–[8].

Recently CALGB 9581 study developed an observational data set of uniformly staged and treated patients describing the prognosis of untreated stage II disease and showing a 5-year all-cause OS of 0.86, with a 5-year disease-specific OS of 0.93. [31]. This notable OS and the low probability of disease-related death could enable the demonstration of a benefit from any adjuvant therapy in stage II colon cancer without accruing thousands of patients.

According to the most important guidelines, some clinical and pathologic prognostic factors (ie, intestinal perforation/obstruction, pathologic stage T4, presence of lymphatic/vascular/perineural invasion, high tumor grade, less than 12 nodes examined) can identify a minority of patients with stage II disease who have higher recurrence risk and who could benefit from adjuvant chemotherapy in current clinical practice.

The Quick and Simple and Reliable (QUASAR) study appreciated that adjuvant chemotherapy with fluorouracil (FU) plus leucovorin (LV) produces an absolute improvement in survival of 3.6% in stage II colon cancer, which should however be balanced with its toxicity, including toxic deaths (approximately 0.5%) [9].This narrow therapeutic index makes it extremely important to select those patients who reserve the adjuvant treatment.

Some recent studies suggest that in patients with stage II disease a deficiency in DNA mismatch repair (dMMR) protein expression and high microsatellite instability (MSI-H) are markers of a more favourable outcome. The inactivation of a DNA mismatch repair (MMR) gene by germline mutation, as in Lynch Syndrome, or more frequently, by sporadic transcriptional silencing results in deficient function of the MMR system and an accumulation of errors in DNA within microsatellites that is termed MSI.

The dMMR was predominantly seen in women, especially older women, compared with men, and in parts of the colon proximal to the splenic flexure, including the transverse colon [29]–[30]. Its detection can identify a subset of stage II colon cancer patients (10%–15%) in whom the benefits of chemotherapy are not sufficiently high to warrant further treatment because of a very low likelihood of recurrence and an absolute benefit from chemotherapy of 1%–2%. [17]–[18], [25]–[27], [32].

Nevertheless, in contrast to previous observations, the availability of more cumulative data from retrospective analyses revealed that MMR status can not be used to predict response to fluoropyrimidine therapy in stage II colon cancer [33].

Although genomic signatures are a rapidly emerging field and have a potentially high prognostic value, none of these signatures is ready for clinical use and is currently predictive for guiding decision on adjuvant treatment in stage II colon cancer [34]–[35].

In our study we analysed patients with T3N0 colon cancer dividing them into two groups of risk based on the presence of at least one of the aforementioned poor prognostic factors (with the esclusion of pathologic T4 and perforated tumors, for which is known the benefit of adjuvant chemotherapy) to evaluate if adjuvant chemotherapy after surgery could improve their prognosis.

As previously reported we confirmed the improvement in RFS and in DFS with the use of adjuvant chemotherapy in high risk stage II patients and showed a statistically significant benefit also in the low risk group in which the 5-years RFS was 87.3% in treated patients and 74.7% in non-treated patients (p 0.03) and DFS was 86.4% in treated patients and 72.4% in patients who underwent to surgery alone (p 0.04).

At multivariate analysis the presence of symptoms at initial diagnosis, sex, treatment and site were confirmed as prognostic factors for RFS, while adjuvant chemotherapy was prognostic factors for all the end-points. Patient's age had prognostic value for overall survival.

In several studies both bowel occlusion and tumor perforation have been identified as prognostic poor factors for the speading of tumor cell in the blood flow and the seeding of tumor cells in the peritoneal cavity respectively [28].

A survival benefit for patients with an extensive examination of lymph nodes for stage II colon cancers was also demonstrated and an adequate resection of lymph nodes should be performed in order to control local recurrence as well [16], [36]. The most important American and European guidelines demand at least 12 lymph nodes to be sampled and examined to accurately determine the stage of colon cancer [1], [6], [16]. The relationship between lymph node retrieved and survival is however not completely understood because no proof that distant metastases can derive from lymph node metastases has so far yielded [37].

In a recent prospective study Santos and coll. confirmed pT4 and lymphatic, venous, or perineural invasion as significant poor prognostic factors for RFS in 432 patients with stage II colon cancer. Differently from our findings the authors also showed a favourable role of female gender in their series [38].

In our retrospective study we excluded from the analysis pT4 and the lack of some pathologic information could not allow to recognise vascular and/or perineural invasion as prognostic negative factors.

Even though toxicity data were not analysed in this report, our data suggest an effective role of adjuvant chemotherapy also in low risk stage II colon cancer: new molecular prognostic factors as dMMR and MSI-I may identify the patients that can really benefit from adjuvant treatment; gene signature represents a potential prognostic biomarker for patients with stage II colon cancer and its effective role is currently tested in ongoing prospective clinical trials.

## References

[1] Jonker DJ, Spithoff K, Maroun J (2011) Adjuvant Systemic Chemotherapy for Stage II and III Colon Cancer after Complete Resection: An Updated Practice Guideline. Clinical Oncology 23 (5) 314–322. 21397476 10.1016/j.clon.2011.02.010

[2] Sato H, Maeda K, Sugihara K, Mochizuki H, Kotake K, et al (2011) High-Risk Stage II Colon Cancer After Curative Resection. Journal of Surgical Oncology 10.1002/jso.21914 21416472

[3] Stocchi L, Fazio VW, Lavery I, Hammel J (2011) Individual surgeon, pathologist and other factors affecting lymph node harvest in stage II colon carcinoma. Is a minimum of 12 examined lymph nodes sufficient? Ann Surg oncol 18: 405–12. 20839064 10.1245/s10434-010-1308-5

[4] NIH Consensus Conference (1990) Adjuvant therapy for patients with colon and rectal cancer. JAMA 264: 1444–50. 2202842

[5] Otchy D, Hyman NH, Simmang C, Anthony T, Buie WD, et al (2004) Practice parameters for colon cancer. Dis Colon rectum 47: 1269–84. 15484340 10.1007/s10350-004-0598-8

[6] National Comprehensive Cancer Network guidelines (2012) version 3. Available: http://www.nccn.org. Accessed 2012 Sept.

[7] Compton CC, Fielding LP, Burgart LJ, Conley B, Cooper HS, et al (2000) Prognostic factors in colorectal cancer. College of American Pathologist Consensus Statement 1999. Arch Pathol Lab Med 124: 979–994. 10888773 10.5858/2000-124-0979-PFICC

[8] Benson AB III, Schrag D, Somerfield MR, Cohen AM, Figueredo AT, et al (2004) American Society of Clinical Oncology recommendations on adjuvant chemotherapy for stage II colon cancer. J Clin Oncol 22: 3408–3419. 15199089 10.1200/JCO.2004.05.063

[9] QUASAR Collaborative Group (2007) Adjuvant chemotherapy versus observation in patients with colorectal cancer: a randomized study. Lancet 370: 2020–2029. 18083404 10.1016/S0140-6736(07)61866-2

[10] Kuebler JP, Wieand HS, O'Connell MJ, Smith RE, Colangelo L, et al (2007) Oxaliplatin combined with weekly bolus fliorouracil and leucovorin as surgical adjuvant chemotherapy for stage II and III colon cancer: results from NSAB P C-07. J Clin Oncol 25: 2198–2204. 17470851 10.1200/JCO.2006.08.2974

[11] Andre T, Boni C, Navarro M, Tabernero J, Hickish T, et al (2009) Improved overall survival with oxaliplatin, fluorouracil, and leucovorin as adjuvant treatment in stage II or III colon cancer in the MOSAIC trial. J Clin Oncol 27: 3109–3116. 19451431 10.1200/JCO.2008.20.6771

[12] Gertler R, Rosenberg R, Schuster T, Friess H (2009) Defining a high-risk subgroup with colon cancer stages I and II for possible adjuvant therapy. Eur J Cancer 45: 2992–2999. 19682890 10.1016/j.ejca.2009.07.008

[13] Figueredo A, Charette ML, Maroun J, Brouwers MC, Zuraw L (2004) Adjuvant therapy for stage II colon cancer: a systematic review from the Cancer Care Ontario Program in Evidence-based Care's Gastrointestinal Cancer Disease Site Group. J Clin Oncol 22 (16) 3395–3407. 15199087 10.1200/JCO.2004.03.087

[14] Figueredo A, Coombes ME, Mukherjee S (2008) Adjuvant therapy for completely resected stage II colon cancer. Cochrane Database Syst Rev Jul 16 (3) CD005390. 10.1002/14651858.CD005390.pub2 PMC8885310 18646127

[15] Quah HM, Chou JF, Gonen M, Jinru S, Schrag D, et al (2008) Identification of patients with high risk stage II colon cancer for adjuvant chemotherapy. Dis Colon Rectum 51: 503–507. 18322753 10.1007/s10350-008-9246-z

[16] Sarli L, Bader G, Iusco D, Salvemini C, Di Mauro D, et al (2005) Number of lymph nodes examined and prognosis of TNM stage II colon cancer. Eur J Cancer 41: 272–279. 15661553 10.1016/j.ejca.2004.10.010

[17] Ribic CM, Sargent DJ, Moore MJ, Thibodeau SN, French AJ, et al (2003) Tumor microsatellite-instability status as a predictor of benefit from fluorouracil-based adjuvant chemotherapy for colon cancer. N Engl J Med 349: 247–257. 12867608 10.1056/NEJMoa022289 PMC3584639

[18] Sargent DJ, Marsoni S, Monges J, Thibodeau SN, Labianca R, et al (2010) Defective mismatch repair as a predictive marker for lack of efficacy of fluorouracil-based adjuvant therapy in colon cancer. J Clin Oncol 28: 3219–3226. 20498393 10.1200/JCO.2009.27.1825 PMC2903323

[19] Schrag D, Rifas-Shiman S, Saltz L, Bach PB, Begg CB, et al (2002) Adjuvant chemotherapy use for medicare beneficiaries with stage II colon cancer. J Clin Oncol 19: 3999–4005. 10.1200/JCO.2002.11.084 12351597

[20] O'Connor ES, Greenblatt DY, LoConte NK, Gangnon RE, Liou J, et al (2011) Adjuvant Chemotherapy for Stage II Colon Cancer With Poor Prognostic Features. J Clin Oncol 29: 3381–3388. 21788561 10.1200/JCO.2010.34.3426 PMC3164243

[21] International Multicentre Pooled Analysis of B2 Colon Cancer Trials (IMPACT B2) Investigators (1999) Efficacy of adjuvant fluorouracil and folinic acid in B2 colon cancer. J Clin Oncol 17: 1356–1363. 10334519

[22] Gill S, Loprinzi CL, Sargent DJ, Thomé SD, Alberts SR, et al (2004) Pooled analysis of fluorouracil-based adjuvant therapy for stage II and III colon cancer: Who benefits and by how much? J Clin Oncol 22: 1797–1806. 15067028 10.1200/JCO.2004.09.059

[23] Sargent D, Sobrero A, Grothey A, O'Connell MJ, Buyse M, et al (2009) Evidence for cure by adjuvant therapy in colon cancer: Observations based on individual patient data from 20,898 patients on 18 randomized trials. J Clin Oncol 27: 872–877. 19124803 10.1200/JCO.2008.19.5362 PMC2738431

[24] Le Voyer TE, Sigurdson ER, Hanlon AL, Mayer RJ, Macdonald JS, et al (2003) Colon cancer survival is associated with increasing number of lymph nodes analyzed: A secondary survey of intergroup trial INT-0089. J Clin Oncol 21: 2912–2919. 12885809 10.1200/JCO.2003.05.062

[25] Weisenberger DJ, Siegmund KD, Campan M, Young J, Long T, et al (2006) CpG island methylator phenotype underlies sporadic microsatellite instability and is tightly associated with BRAF mutation in colorectal cancer. Nat Genet 38: 787–793. 16804544 10.1038/ng1834

[26] Sinicrope FA, Sargent DJ (2009) Clinical implications of microsatellite instability in sporadic colon cancers. Curr Opin Oncol 21: 369–373. 19444104 10.1097/CCO.0b013e32832c94bd PMC3761884

[27] Kim GP, Colangelo LH, Wieand HS, Paik S, Kirsch IR, et al (2007) Prognostic and predictive roles of high-degree microsatellite instability in colon cancer: A National Cancer Institute-National Surgical Adjuvant Breast and Bowel Project Collaborative Study. J Clin Oncol 25: 767–772. 17228023 10.1200/JCO.2006.05.8172

[28] Schmoll HJ, Van Cutsem E, Stein A, Valentini V, Glimelius B, et al (2012) ESMO Consensus Guidelines for management of patients with colon and rectal cancer. A personalized approach to clinical decision making. Annals of Oncology 23: 2479–2516 10.1093/annonc/mds236 23012255

[29] Sinicrope FA, Sargent DJ (2012) “Molecular Pathways: Microsatellite Instability in Colorectal. Cancer: Prognostic, Predictive, and Therapeutic Implications.”. Clinical Cancer Res 18 (6) 10.1158/1078-0432.CCR-11-1469 PMC3306518 22302899

[30] Poynter JN, Siegmund KD, Weisenberger DJ, Long TI, Thibodeau SN, et al (2008) Colon Cancer Family Registry Investigators. Molecular characterization of MSI-H colorectal cancer by MLHI promoter meth-ylation, immunohistochemistry, and mismatch repair germline muta- tion screening. Cancer Epidemiol Biomarkers Prev 17: 3208–3215. 18990764 10.1158/1055-9965.EPI-08-0512 PMC2628332

[31] Niedzwiecki D, Bertagnolli MM, Warren RS, Compton CC, Kemeny NE, et al (2011) Documenting the Natural History of Patients with Resected Stage II Adenocarcinoma of the Colon after Random Assignment to Adjuvant Treatment with Edrecolomab or Observation: Results From CALGB 9581. JCO 29: 3146–3152. 10.1200/JCO.2010.32.5357 PMC3157980 21747085

[32] Roth AD, Delorenzi M, Tejpar S, Yan P, Klingbiel D, et al (2012) Integrated Analysis of Molecular and Clinical Prognostic Factors in Stage II/III Colon Cancer. J Natl Cancer Inst 104 (21) 1635–1646 10.1093/jnci/djs427 23104212

[33] Hutchins G, Southward K, Handley K, Magill L, Beaumont C, et al (2011) Value of Mismatch Repair, KRAS, and BRAF Mutations in Predicting Recurrence and Benefits From Chemotherapy in Colorectal Cancer. JCO 29: 1261–1270. 10.1200/JCO.2010.30.1366 21383284

[34] Salazar R, Roepman P, Capella G, Moreno V, Simon I, et al (2011) Gene expression signature to improve prognosis prediction of stage II and III colorectal cancer. J Clin Oncol 29: 17–24. 21098318 10.1200/JCO.2010.30.1077

[35] Gray RG, Quirke P, Handley K, Lopatin M, Magill L, et al (2011) Validation study of a quantitative multigene reverse transcriptase-polymerase chain reaction assay for assessment of recurrence risk in patients with stage II colon cancer. J Clin Oncol 29: 4611–4619. 22067390 10.1200/JCO.2010.32.8732

[36] Chang GJ, Rodriguez-Bigas MA, Skibber JM, Moyer VA (2007) Lymph node evaluation and survival after curative resection of colon cancer: systematic review. J Natl Cancer Inst 99 (6) 433–441. 17374833 10.1093/jnci/djk092

[37] Laubert T, Habermann JK, Hemmelmann C, Kleemann M, Oevermann E, et al (2012) Metachronous metastasis- and survival-analysis show prognostic importance of lymphadenectomy for colon carcinomas. BMC Gastroenterology 12: 24 Available: http://www.biomedcentral.com/1471-230X/12/24. accessed 2013 Nov 14. 22443372 10.1186/1471-230X-12-24 PMC3349572

[38] Santos C, López-Doriga A, Navarro M, Mateo J, Biondo S, et al (2012) Clinico-pathological risk factors of stage II colon cancer: results of a prospective study. Colorectal Dis 10.1111/codi.12028 22974322

